# Experimental Investigation on the Impact of Coal Fines Migration on Pores and Permeability of Cataclastic Coal

**DOI:** 10.1021/acsomega.3c03433

**Published:** 2023-08-17

**Authors:** Tiancheng Xie, Yingchun Wei, Ziliang Liu, Biao Li, Daiyong Cao, Anmin Wang

**Affiliations:** † College of Geoscience and Surveying Engineering, China University of Mining and Technology-Beijing, Beijing 100083, China; ‡ State Key Laboratory of Coal Resources and Safe Mining, China University of Mining and Technology-Beijing, Beijing 100083, China

## Abstract

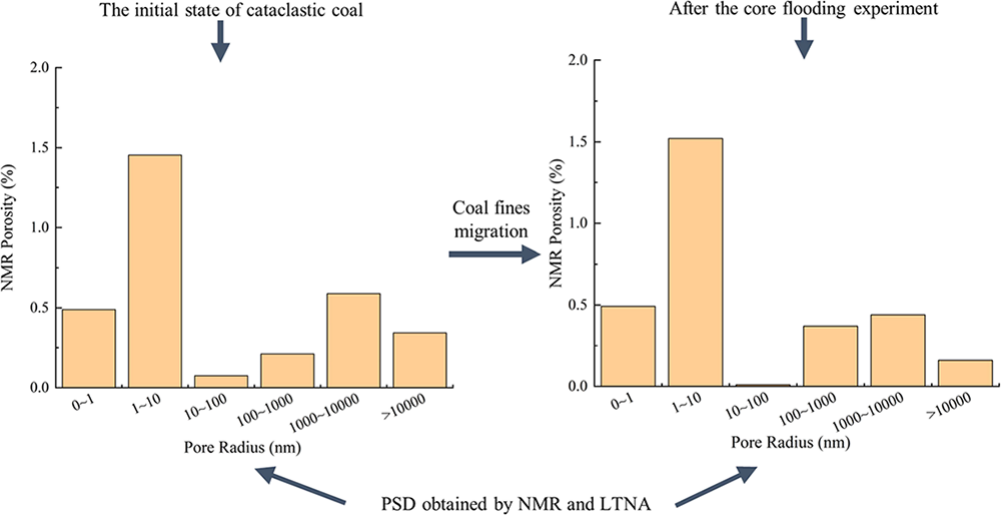

During the production process of coalbed methane, the generation and migration of coal fines can obstruct fractures in coal reservoirs and reduce their permeability. In order to investigate the effects of coal fines migration on the porosity and permeability of coal reservoirs, we conducted core water flooding experiments, low-field nuclear magnetic resonance (NMR), and low-temperature N_2_ adsorption experiments to study the variations in porosity and permeability of cataclastic coal during coal fines migration and the impact of coal fines migration on porosity and permeability. The experimental results reveal that the initial porosity ratio of cataclastic coal exhibits the characteristics of micropore > macropore > transitional pore > mesopore, with the pore types being predominantly fissured. The porosity of pores larger than 1000 nm and those larger than 10,000 nm exhibit consistent trends before and after water flooding, indicating that the blockage or unblocking of pores with radius larger than 10,000 nm by coal fines can also cause blockage or unblocking of some interconnected macropore. The early stage of flooding is the main period for coal fines migration and production in cataclastic coal, during which the mass concentration of coal fines production is higher and some macropores and fractures become blocked, resulting in a larger decrease in porosity. The higher the initial permeability of cataclastic coal samples with a larger end-face fracture density, the more similar the variations in porosity and permeability of pores larger than 10,000 nm during the flooding experiment, indicating that coal fines mainly block interconnected pores and fractures with radius larger than 10,000 nm through migration, thereby reducing permeability. This study provides a theoretical basis for the efficient production of coalbed methane.

## 1 Introduction

The issue of coal fines production has long been severely affecting the stable production of coalbed methane. During the generation and migration process, coal fines can block coal reservoir fractures, causing a decrease in reservoir permeability and blocking the bottom-hole screen pipes and pump inlets, leading to production failures.^1−3^ The production of coal fines during the coalbed methane production process is controlled by engineering and geological factors,^4−8^ with different production stages exhibiting varying coal fines production patterns.^9−11^ The drilling process, including the grinding of coal seams by drilling tools, the erosion of coal reservoirs by water flow during drainage, and the abrasion of coal reservoirs by fracturing fluids or proppants during fracturing, can result in a large amount of coal fines.^12−15^ In addition, stress changes induced by coalbed methane production can also lead to shear failure in the coal reservoir, resulting in the generation of coal fines.^16^ Geological structures and coal quality also affect the production of coal fines;^5,6^ tectonic coal, which forms under tectonic deformation, has a complex pore structure and features low strength and low permeability, making it prone to damage and the production of large amounts of coal fines during the production process^17−20^ and causing reservoir damage and seriously affect production.

Simulation experiments further demonstrate that various factors can impact coal fines migration, leading to a decrease in permeability, such as pressure,^21−23^ flow rate,^24−27^ pressure gradient,^28−30^ and coal structure.^31−33^ Excessive pressure can cause the closure of pore–fracture channels for coal fines migration, making it difficult for coal fines to be produced and leading to a decrease in permeability.^34^ The velocity-sensitive effect induced by flow rate can result in a large amount of coal fines production and a significant decrease in permeability.^35^ The larger the pressure gradient, the wider the particle size range of coal fines produced, and there exist a critical value that causes a significant decrease in permeability and a large amount of coal fines production. The coal structure and mineral composition can affect the characteristics of produced coal fines, such as particle size and clay mineral content, which in turn further influence the permeability of coal rocks.^36−40^ It has also been pointed out that higher temperatures will affect the migration of fine particles and lead to the decrease of permeability.^41,42^ These research findings mainly focus on the production patterns of coal fines under different conditions and the variation of permeability due to coal fines migration, but the impact of coal fines migration on pore changes and the mechanism of permeability variation have not been considered. Moreover, most experiments use raw coal samples or briquette samples made from coal fines, with no research specifically targeting coal fine migration in cataclastic coal.

To study the pore change patterns during coal fines migration, it is necessary to characterize the pore structure of the coal. Due to the influence of tectonic-thermal evolution on cataclastic coal, its strength is relatively low,^43^ and traditional mercury intrusion methods can easily damage its pore structure, rendering the measured results unable to accurately reflect the original pore structure characteristics and incapable of accurately measuring micropore information.^44,45^ Low-temperature N_2_ adsorption (LTNA) allows for nondestructive measurements but mainly targets information on certain micropores.^45−47^ Nuclear magnetic resonance (NMR) technology can nondestructively measure fluid information in the pores of saturated samples, thus obtaining the full-scale pore structure characteristics of the samples.^48,49^ However, using NMR technology directly cannot obtain the pore size distribution (PSD) of coal samples,^50^ and other means must be combined to assist in calculating conversion factors, such as the mercury intrusion porosimetry,^51^ the surface-to-volume ratio (SVR),^52^ and the *T*_2c_-cutoff value.^53^ In comparison, the combined method of low-field NMR and combining the SVR data from LTNA experiments can more accurately obtain the pore structure characteristics of cataclastic coal without damaging the original pore structure of the coal sample.

Based on previous research, this study uses physical simulation experiments of coal fines migration and its impact on pore permeability in cataclastic coal reservoirs, combined with low-field NMR and LTNA methods, to further investigate the influence of coal fines migration on the pore–fracture structure and permeability of cataclastic coal as well as to explore the relationship between pore–fracture changes and permeability during the coal fines migration process.

## 2 Experimental Methodology

### 2.1 Sample Preparation

Cataclastic coal samples were collected from the Shanxi Formation No. 3 coal seam in the Hancheng mining area. The macroscopic characteristics of the coal rock are as follows: the original stratified structure is relatively intact; the fracture surface is jagged; it is easily broken and can exhibit flaky and granular exfoliation, turning into powder when rubbed by hand; and the fractures are well-developed. Petrographic and chemical analyses were performed on the collected cataclastic coal samples, with the results of the proximate analysis and ultimate analysis shown in Tables 1 and 2. The maximum vitrinite reflectance of the sample under oil immersion is 1.64%, and the maceral and mineral composition test results are shown in Table 3. Three cylindrical coal core samples with a diameter of 25 mm and a length of 50 mm were drilled from the collected coal samples for NMR analysis and core flooding experiments. An end with well-developed fractures was selected as the outlet to ensure stable production of coal fines. Prior to the experiments, the specific surface area and pore volume of the cataclastic coal samples were measured by LTNA, resulting in values of 0.261 m^2^/g and 5.56 × 10^–4^ mL/g, respectively.

**Table 1:** Results of the Proximate Analysis

	(%)
moisture	0.56
ash	16.05
volatile matter	11.89
fixed carbon	71.5

**Table 2:** Results of the Ultimate Analysis

	(%)
carbon	1.26
hydrogen	77.01
nitrogen	4.26
oxygen	2.17

**Table 3:** Results of the Maceral and Mineral Composition Test

	(%)
vitrinite	74.12
inertinite	14.7
mineral	11.18

### 2.2 Core Flooding Experiment

To simulate the production and migration of coal fines in cataclastic coal, core water flooding experiments were conducted using an LYD32 core flow device. The device can adjust the confining pressure through annular pressure regulation and can set the flow rate to stably inject fluid into the coal core at a constant flow rate. The experimental parameters, such as the pressure and flow rate, were recorded to calculate permeability. Three groups of core flow experiments were conducted using the device, with coal samples A, B, and C subjected to different confining pressures and flow rate conditions: 0.5 mL/min and 5 MPa, 1 mL/min and 5 MPa, and 1 mL/min and 3 MPa, respectively. Deionized water was used as the flooding fluid.

**Figure 1:**
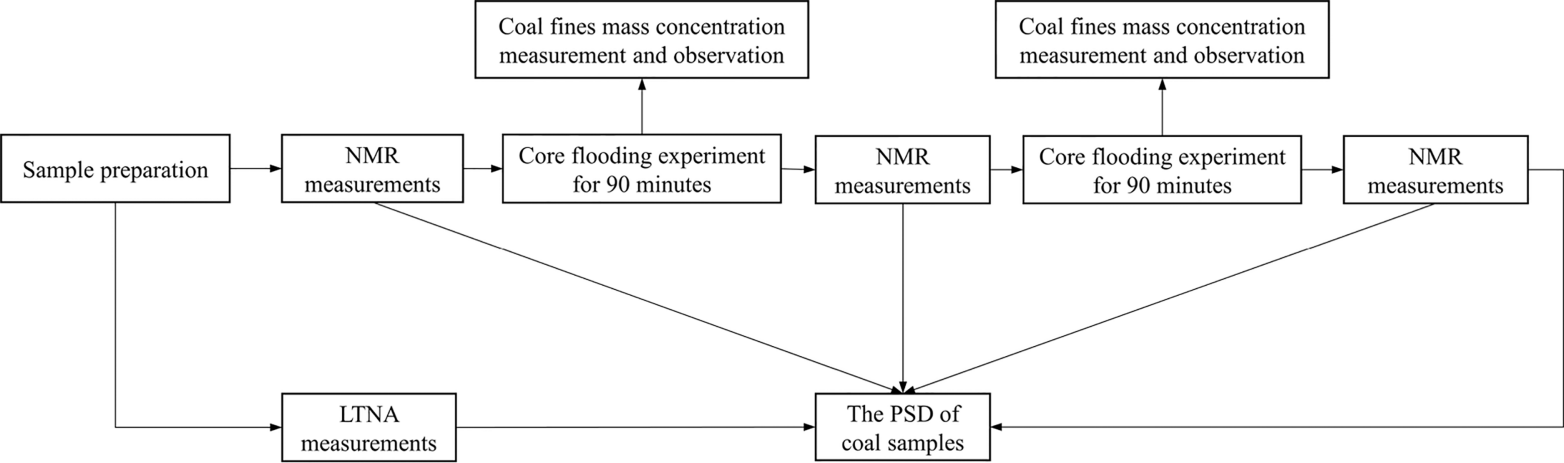
Entire experimental procedure.

After 90 and 180 min of the core flooding experiment, the coal fines containing solution was collected, and the coal fines yield was determined by collecting and drying the solution on quantitative filter paper. The produced coal fines were then coated with gold and observed under a scanning electron microscope (SEM) to analyze their particle size and morphological characteristics (Figure 1).

### 2.3 Nuclear Magnetic Resonance

NMR experiments were conducted before the core flooding experiment, after 90 min of water flooding, and after 180 min of water flooding to analyze the changes in the pore–fracture system of the cataclastic coal before and during the coal fines migration. Before the NMR analysis, the cylindrical coal core samples were vacuumed for 2 h in a vacuum pressure saturation device and then pressurized for 24 h at 15 MPa for water saturation. The saturated samples were used for NMR measurements, which were performed using a MiniMR NMR analyzer combined with NMR core analysis measurement software, measuring the porosity and the *T*_2_ spectrum of the water-saturated core samples through the Carr-Purcell-Meiboom-Gill (CPMG) sequence. The main NMR experimental parameters included an echo time of 0.1 ms, echo numbers of 10,000, a relaxation delay of 5000 ms, and 64 scan times, measuring the transverse relaxation time of water in the saturated samples using low-field NMR technology. Although NMR can reflect the full-scale pore size characteristics through the *T*_2_ spectrum, it cannot directly determine the specific PSD. In this study, the SVR method was used to combine the cataclastic coal specific surface area and pore volume obtained from LTNA with the low-field NMR *T*_2_ results to calculate the values of surface relaxivity ρ_2_ of the cataclastic coal samples and then obtain the specific PSD of the cataclastic coal samples. Under the conditions of low-field NMR, the transverse relaxation time of fluid in coal can be expressed by the following formula:^48^
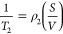
1ρ_2_ is the value of surface relaxivity; *S* is the surface area of the sample; *V* is the pore volume of the sample, simplifying the pore shape in coal and the above equation can be expressed as
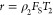
2

In the formula, *F*_S_ represents the pore shape factor. For fissured, columnar, and spherical pores, *F*_S_ takes values of 1, 2, and 3, respectively. For the same type of coal, ρ_2_ is a constant, so the PSD of the coal can be determined based on the *T*_2_ values. The results of LTNA indicate that the pore morphology of the cataclastic coal samples is relatively complex, with the possibility of fissured, columnar, and spherical pores existing. Therefore, the PSDs were calculated for each of the three shape factors and compared with the PSDs obtained from LTNA. The values of surface relaxivity were calculated using the SVR method, and the logarithmic mean value *T*_2LM_ of the *T*_2_ spectrum was computed. Then, using the BET model pore area and BJH model pore volume obtained from the LTNA results, the values of surface relaxivity of the cataclastic coal samples were calculated in conjunction with eq 1. The formula for calculating *T*_2LM_ is as follows:^54^
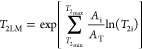
3where *A*_i_ refers to the amplitude at *T*_2i_; *A*_T_ refers to the total amplitude of the NMR spectrum; *T*_2i_ is the individual value of *T*_2_, unit ms, and finally the value of ρ_2_ can be calculated by combining with the relaxation time expression of the pore fluid (eq 1).

## 3 Results

### 3.1 Characterization of the Produced Fines

In each core flooding experiment, quantitative filter paper was used to collect the coal fines produced from the cataclastic coal and weighed. The obtained coal fines concentrations are shown in Table 4. Compared to the first 90 min of water flooding, the mass concentration of coal fines produced in coal samples A, B, and C decreased by 74, 54, and 87% after an additional 90 min of water flooding, respectively. The average mass concentrations of coal fines produced during the entire water flooding process for coal samples A–C were 30.38, 12.92, and 15.35 mg L^–1^, respectively. Overall, after the additional 90 min of water flooding, the reduction in coal fines production for all three samples was more than 50%.

**Table 4:** Mass Concentration of Coal Fines Produced from Different Cataclastic Coal Samples

sample	water flooding time (min)	mass concentration (mg/L)
A	0–90	48.15
	90–180	12.6
B	0–90	17.71
	90–180	8.13
C	0–90	27.08
	90–180	3.62

**Figure 2:**
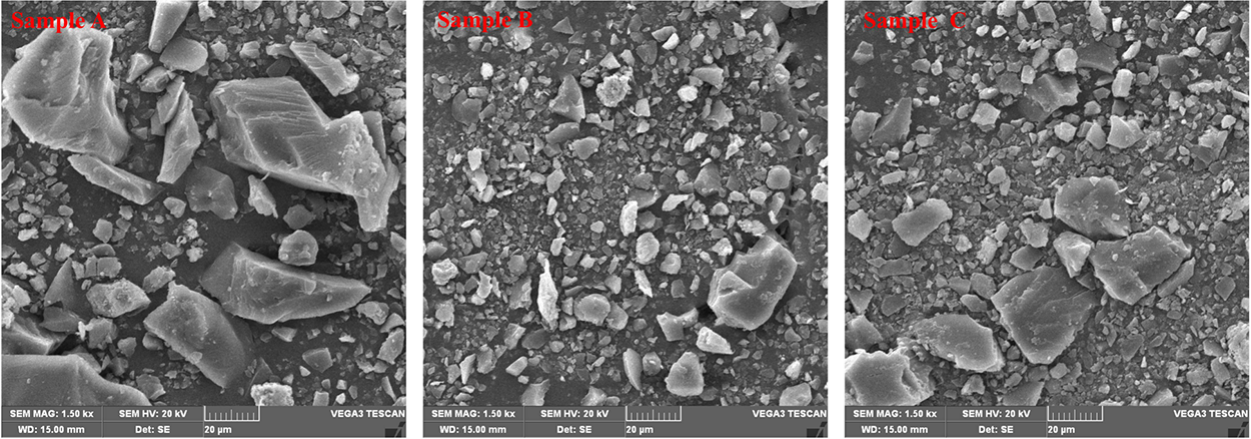
SEM images of coal fines produced by each coal sample.

By observing the produced coal fines under a microscope, the characteristics of the coal fines produced from the three cataclastic coal samples were quite similar. Larger coal fines were mostly angular or subangular in shape, while some smaller coal fine particles exhibited subrounded characteristics. The particle size distribution range was relatively wide, with some larger coal fines having a particle size larger than 1000 nm, and even some coal fines with particle sizes exceeding 10,000 nm (Figure 2).

### 3.2 Permeability

**Figure 3:**
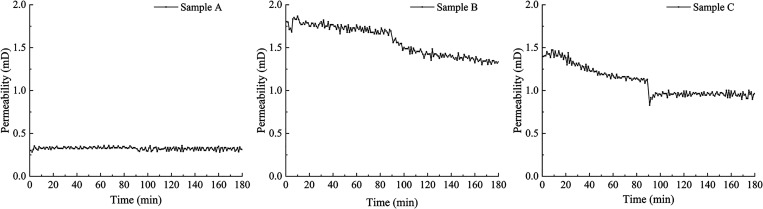
Permeability changes of cataclastic coal samples during the core flooding experiment.

The real-time changes in permeability were calculated by monitoring the differential pressure at the inlet and outlet of the core flooding device and the mass of the effluent (Figure 3). The average differential pressures are shown in Table 5. The average permeability of coal sample A during the entire water flooding process was 0.33 mD. The average permeability of the coal sample during the first 90 min was 0.33 mD. After an additional 90 min of water flooding, the average permeability of the coal sample decreased to 0.32 mD, a decline of 4%. The permeability of the coal sample remained stable throughout the experiment, with a larger fluctuation in the last 90 min. The average permeability of coal sample B during the entire water flooding process was 1.58 mD. The average permeability of the coal sample during the first 1.5 h was 1.74 mD. After an additional 90 min of water flooding, the average permeability of the coal sample decreased to 1.41 mD, a decline of 19%. The permeability of the coal sample exhibited a downward trend throughout the experiment. The average permeability of coal sample C during the entire water flooding process was 1.10 mD. The average permeability of the coal sample during the first 90 min was 1.25 mD. After an additional 90 min of water flooding, the average permeability of the coal sample decreased to 0.95 mD, a decline of 24%.

**Table 5:** Average Permeability and Inlet/Outlet Differential Pressure of Coal Samples

sample	water flooding time (min)	average inlet/outlet differential pressure (MPa)
A	0–90	2.38
	90–180	2.49
B	0–90	0.94
	90–180	1.14
C	0–90	1.33
	90–180	1.73

### 3.3 Result of NMR Measurement

**Figure 4:**
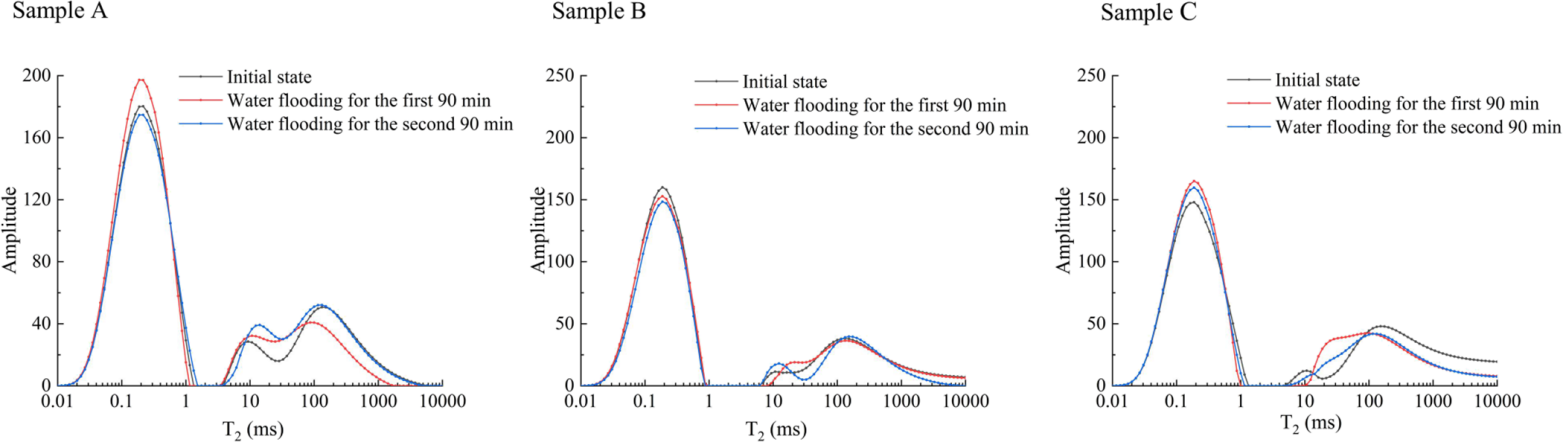
NMR T_2_ spectra of cataclastic coal samples.

The NMR results for the three cataclastic coal samples are shown in Figure 4. The NMR spectra of three cataclastic coal samples were similar, and all have three distinct peaks. The relaxation times for these peaks were approximately distributed within the ranges of 0.05–1 ms, 5–20 ms, and >100 ms. These three peaks corresponded to adsorption pores (including micropores and transitional pores, pore size <100 nm), seepage pores (including mesopores and macropores, pore size 100–100,000 nm), and fractures (pore size >100,000 nm).^53,55^ These results indicated that the porosity of the cataclastic coal samples was dominated by micropores and transitional pores, followed by fractures, while mesopores and macropores appeared to be relatively underdeveloped. The NMR porosity of the three coal samples is between 2.96 and 3.30%. Notably, the changes in the NMR *T*_2_ spectrum peak shapes for the cataclastic coal samples before and after water flooding were small. The changes in the abscissa (relaxation time) represented by the three peaks are not significant, and the changes in the ordinate (amplitude) are also small (Figure 4). This indicates that the migration of coal fines during the experiment may cause fluctuations in the pore content but will not lead to a transformation in the overall pore structure of the cataclastic coal. By using all *T*_2_ data obtained from low-field NMR combined with the surface area and pore volume obtained from LTNA, the average ρ_2_ value of the three samples calculated using formulas 1 and 3 is 1.35 μm/s. By using the same method but only using the data representing the adsorption pore peak in low-field NMR, the average ρ_2_ of the three samples is 11.66 μm/s.

## 4 Discussion

### 4.1 PSD of Cataclastic Coal

**Figure 5:**
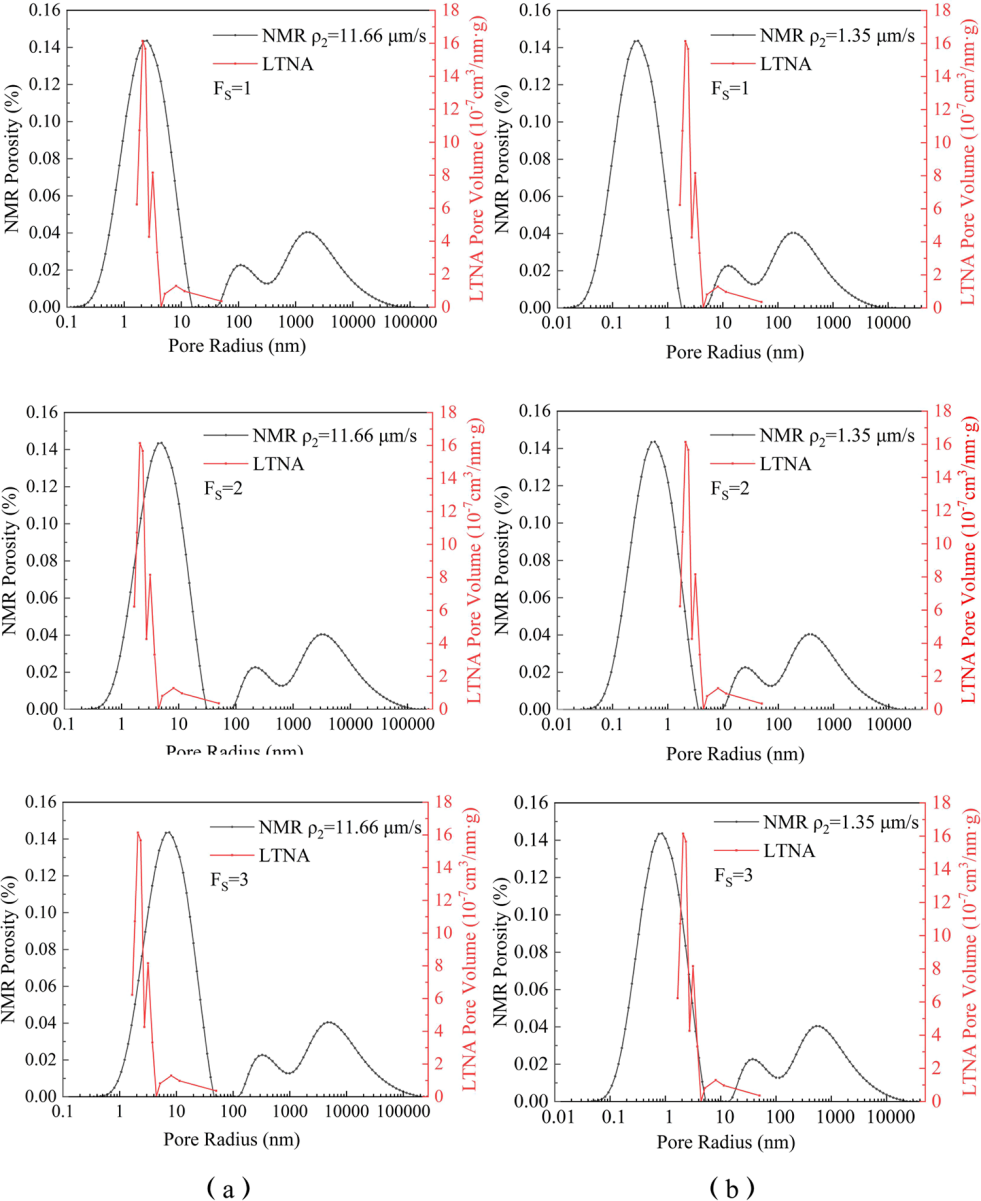
NMR PSDs of sample A obtained from ρ_2_ transformations using *T*_2_ data representing the microtransitional pore peaks (a) and NMR PSDs of sample A obtained from ρ_2_ transformations using all *T*_2_ data (b).

Due to the fact that LTNA mainly detects smaller pores (*r* < 100 nm), while the *T*_2_ spectra data obtained from NMR represent the pore size information on all sizes, this study, based on the previous research on coal NMR peak classification, uses the data representing the adsorption pore peak (*r* < 100 nm) in the *T*_2_ spectra to calculate the values of surface relaxivity using the SVR method. This allowed us to obtain the PSD and compare the results with the PSD obtained using all *T*_2_ data. The ρ_2_ values obtained by these two methods are then used to convert the cataclastic coal NMR *T*_2_ spectra into full-size PSDs, which are compared to the PSD obtained from LTNA (Figure 5).

**Figure 6:**
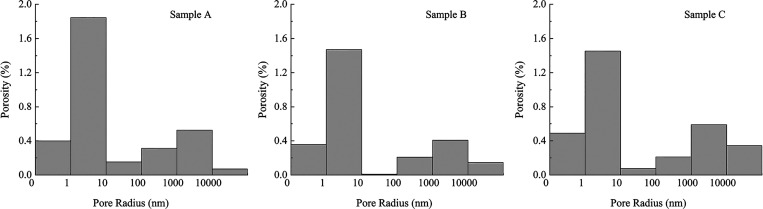
Pore throat distribution of cataclastic coal samples.

The results show that the PSD calculated using only the *T*_2_ spectra data representing the adsorption pores (pore size <100 nm) peak, combined with SVR, is more accurate than that obtained using all *T*_2_ data. When the pore shape factor *F*_S_ = 1, the PSD obtained using ρ_2_ calculated from the adsorption pore peak *T*_2_ values has the highest degree of matching with the PSD, indicating that the primary pore shape in cataclastic coal samples is fissured. The ρ_2_ values obtained were used to calculate the PSD of the three cataclastic coal samples. Subsequently, based on Hodot’s pore classification method, the PSD was transformed into a pore throat distribution with different pore size contents. As shown in Figure 6, the initial pore throat distribution characteristics of the three cataclastic coal samples are essentially the same. Micropores with radius of 0–10 nm have the highest content, with an average proportion of 67%. This is followed by macropores with radius of 1000–10,000 nm, with an average proportion of 17%. The third highest content is mesopores with radius of 100–1000 nm, with an average proportion of 8%. Transitional pores with radius of 10–100 nm have the lowest proportion, with an average of only 2%. After excluding pores larger than 10,000 nm, the overall pore content proportions of the three samples are micropore > macropore > mesopore > transitional pore, which might be a common feature of this type of cataclastic coal pore structure. However, the proportions of pores larger than 10,000 nm vary among the three cataclastic coal samples. In coal sample A, their content is the lowest, lower than transitional pores. In coal sample B, the content is higher than transitional pores but lower than mesopores, while in coal sample C, the content is the highest, higher than mesopores. This indicates that the main distinguishing feature of the pore characteristics in the three cataclastic coal samples is the different proportions of pores larger than 10,000 nm. It further indicated that the pores larger than 10,000 nm are not uniform in the cataclastic coal.

### 4.2 Effect of Coal Fines Migration on the Pore Structure of Cataclastic Coal

**Figure 7:**
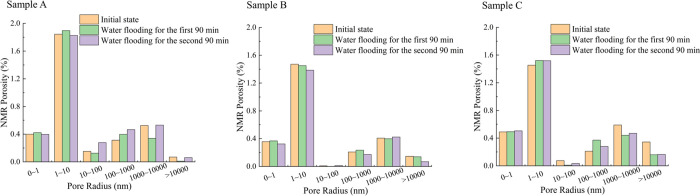
Changes of the pore throat distribution of cataclastic coal samples during the core flooding experiment.

**Figure 8:**
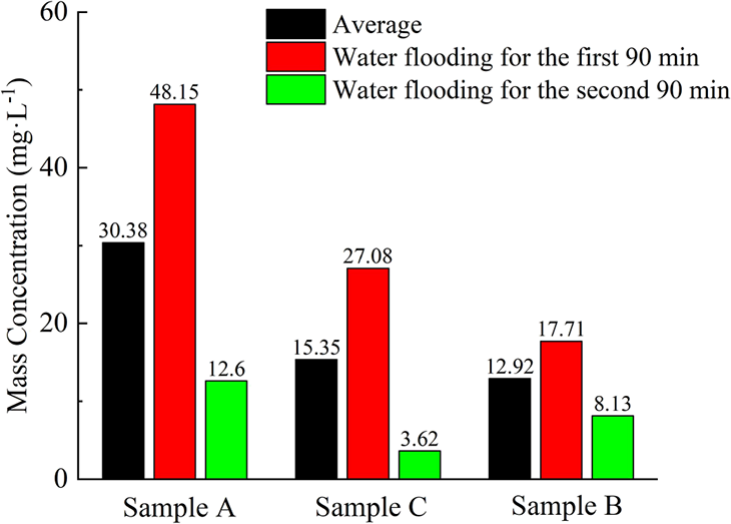
Mass concentration of coal fines produced by coal samples.

During the water flooding process, the changes in porosity of macropores with radii of 1000–10,000 nm and pores larger than 10,000 nm are relatively similar for all three coal samples (Figure 7). This suggests good connectivity between these two pore size ranges. When pores larger than 10,000 nm are blocked or unblocked by coal fines, some of the macropores with radius of 1000–10,000 nm that are connected to them may also become blocked or unblocked. In addition, SEM observations reveal that the maximum particle size of produced coal fines exceeds 1000 nm, further indicating that pores and fractures with radius larger than 1000 nm are the main pathways for coal fines migration and blockage. After 90 min of flooding experiments, the mass concentration of produced coal fines is much higher than that observed between 90 and 180 min (Figure 8). At the same time, there is a significant decline in pores larger than 10,000 nm in the coal samples after 90 min, indicating that the early stage of the flooding experiment is the primary period of coal fines migration and production. During this time, most of the coal fines in the coal samples migrate under the action of water flooding, with some being discharged through connected pores and fractures, and others blocking part of the pores and fractures, causing damage to permeability. After the interruption and restart of the experiment, there is a significant decrease in the coal fines production quality and concentration, as well as the permeability. This is because prior to the interruption experiment, the coal fines were suspended in the fluid and transported along with the fluid flow. However, after the experiment was interrupted, the fluid flow ceased, causing the coal fines to settle and accumulate, subsequently redistributing within the fracture spaces. Upon restarting the experiment, the settled and accumulated coal fines were more prone to blocking the fractures, leading to subsequent impact on the coal fines production and permeability damage.

### 4.3 Influence of Pore Change of Cataclastic Coal on Permeability during Coal Fines Migration

**Figure 9:**
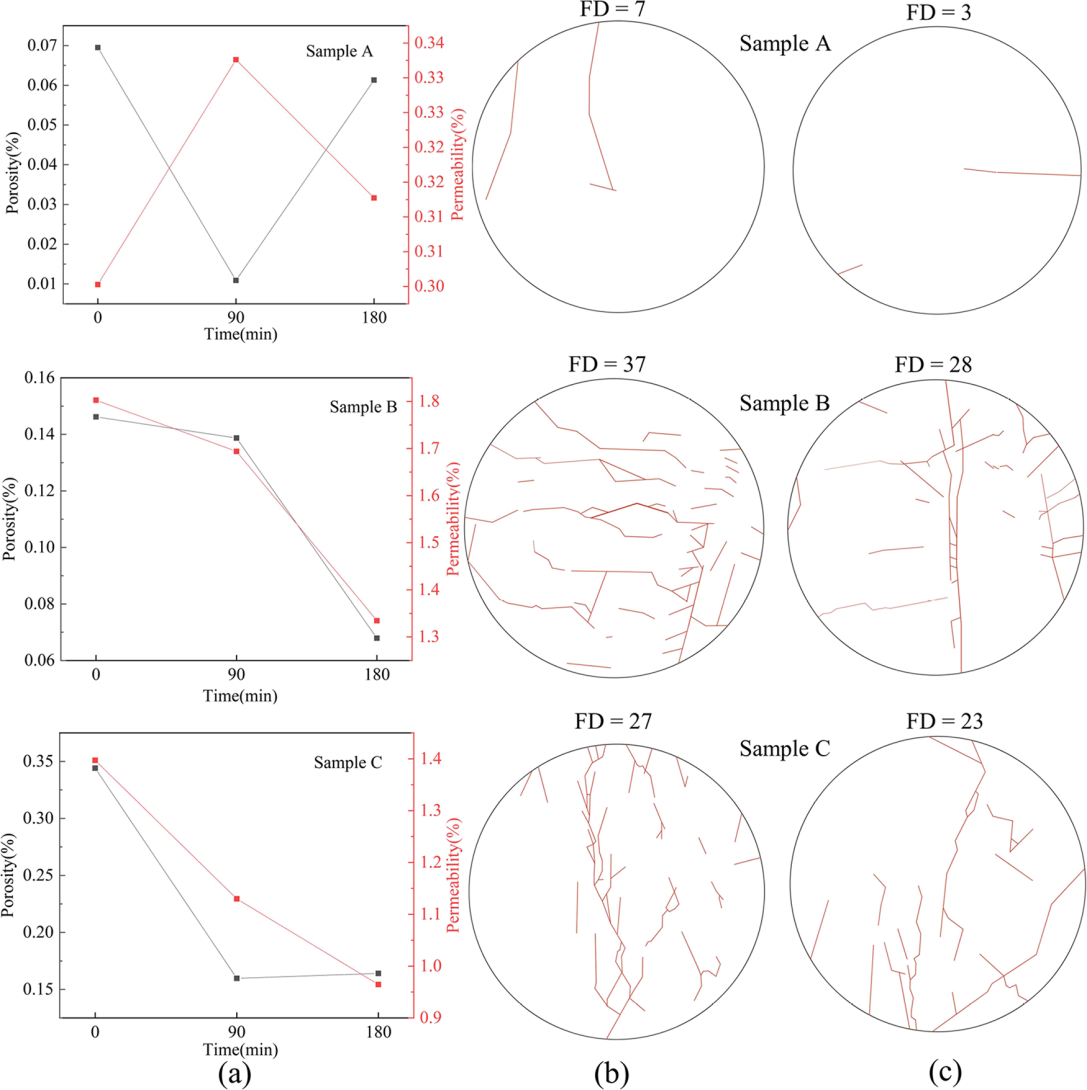
Changes in permeability and porosity of >10,000 nm pores (a) before and after flooding experiments; (b) fractures at outlet ends of coal samples (FD = fracture density, unit: strip/cm); and (c) fractures at inlet ends of coal samples.

The initial porosity of the three coal samples is as follows: coal sample A > coal sample C > coal sample B, while the average permeability is in the order of coal sample B > coal sample C > coal sample A. This indicates that the overall porosity has a relatively small impact on the permeability of cataclastic coal. Previous studies have shown that seepage pores (>100 nm) are important channels for fluid migration, and effective porosity is mainly provided by pores with radius larger than 100 nm.^56^ Additionally, the distribution of natural fractures in coal, as well as their connectivity in the direction of flow, can have a significant impact on effective porosity and permeability.^57−60^ The development of surface fractures in the three cataclastic coal samples is shown in Figure 9. Coal sample A has the lowest fracture density at the inlet and outlet, resulting in the lowest initial permeability. Therefore, under low flow conditions, the differential pressure between the inlet and outlet is much larger than that of coal samples B and C, making it more likely to produce a large amount of coal fines.^28,29^ At the same time, coal fines are difficult to discharge directly from pores and fractures and are more likely to accumulate and block in the bent channels, causing a decrease in permeability.^21^ This further leads to an increase in the differential pressure between the inlet and outlet. When the differential pressure rises to a certain level, it reaches the pressure gradient required for releasing larger particle sizes,^29^ causing some blocked coal fines to become unblocked and increasing the porosity of some pores larger than 10,000 nm. This is reflected in the fluctuation of permeability. Coal sample B has the highest fracture density at the inlet and outlet, leading to a higher initial permeability. Therefore, even when the flow rate is increased, the differential pressure between the inlet and outlet remains the lowest. Under a relatively low-pressure gradient, a stable and small amount of coal fines are produced, and the porosity of >10,000 nm pores decreases slightly due to minor coal fines deposition blockage. The decrease in porosity of some connected pores leads to a drop in permeability. For coal sample C, the fracture density at the inlet and outlet is between that of samples A and B, and the differential pressure between the inlet and outlet is between that of samples B and C. Under the influence of the pressure gradient, coal fines in sample C are more likely to migrate and block than those in sample B, resulting in a higher degree of decline in the porosity of >10,000 nm pores and a larger decrease in permeability.

For coal samples with a higher density of end-face fractures and higher initial permeability (Figure 9), the coal fines can more easily migrate and block the fractures and pores during the water flooding process, causing a decrease in effective porosity and permeability. Simultaneously, coal samples with a higher density of end-face fractures exhibit a more similar trend in terms of porosity and permeability changes for pores larger than 10,000 nm (Figure 9). This indicates that as the density of fractures at both ends of a coal sample increases, the proportion of effective porosity (the content of interconnected pores) for pores larger than 10,000 nm also increases. On the other hand, there is generally poor correlation, or even negative correlation, between permeability and the pores of other sizes, it indicates that coal fines block interconnected pores and fractures with a radius greater than 10,000 nm through migration, leading to a decrease in permeability.

## 5 Conclusions

In order to study the influence of coal fines migration on the pore and permeability of cataclastic coal, a physical simulation experiment of coal fines migration was conducted on cataclastic coal samples. The changes in permeability and coal fines production during the migration process were obtained, and the changes in PSD were determined using NMR and LTNA. The main research findings are as follows:

1. The PSD of cataclastic coal was calculated through LTNA and NMR. When the pore shape factor *F*_S_ is 1, the fitting effect with the PSD obtained by LTNA is the best, indicating that the dominant pore shape in the cataclastic coal samples is fissured. Overall, the proportion of pore contents in cataclastic coal exhibits the following characteristics: micropores > macropores> transitional pores > mesopores.

2. The changes in macropores with radius of 1000–10,000 nm and pores with radius larger than 10,000 nm were consistent before and after water flooding. The blockage or unblocking of pores larger than 10,000 nm by coal fines would cause some interconnected macropores to also become blocked or unblocked. Additionally, some of the produced coal fines have particle sizes larger than 1000 nm, indicating that macropores and fractures with radius larger than 1000 nm are the main channels for coal fines migration and blockage. The production of coal fines is mainly concentrated in the first 90 min of the core flooding experiment, during which the coal fines produced concentration is relatively high and is also the main period for coal fines migration and blockage.

3. The higher the fracture density on the end face of the cataclastic coal sample, the higher the initial permeability. In addition, the more similar the changes in the porosity and permeability of the pores larger than 10,000 nm during the flooding experiment. This suggests that coal fines migrate and block interconnected pores and fractures with radius larger than 10,000 nm, subsequently leading to a decrease in permeability.
